# Graphene-Based Nanosystem for Targeted Delivery of Anti-Sense miRNA-21 on Hepatocellular Carcinoma Cells

**DOI:** 10.3390/ijms27020975

**Published:** 2026-01-19

**Authors:** Paola Trischitta, Paulina Kucharzewska, Barbara Nasiłowska, Wojciech Skrzeczanowski, Rosamaria Pennisi, Maria Teresa Sciortino, Marta Kutwin

**Affiliations:** 1 Department of Chemical, Biological, Pharmaceutical and Environmental Science, University of Messina, Viale Ferdinando Stagno d’Alcontres 31, 98166 Messina, Italy; paola.trischitta@dottorandi.unipg.it (P.T.); rpennisi@unime.it (R.P.); 2 Department of Chemistry, Biology, and Biotechnology, University of Perugia, Via Elce di Sotto 8, 06123 Perugia, Italy; 3 Center of Cellular Immunotherapies, Warsaw University of Life Sciences, 8 Ciszewskiego St., 02-786 Warsaw, Poland; paulina_kucharzewska_siembieda@sggw.edu.pl; 4 Institute of Optoelectronics, Military University of Technology, gen. S. Kaliskiego 2, 00-908 Warsaw, Poland; barbara.nasilowska@wat.edu.pl (B.N.); wojciech.skrzeczanowski@wat.edu.pl (W.S.); 5 Department of Nanobiotechnology, Institute of Biology, Warsaw University of Life Sciences, Ciszewskiego 8, 02-786 Warsaw, Poland

**Keywords:** GO, antisense miRNA-21, cancer, hepatocellular carcinoma, drug delivery

## Abstract

The application of nanotechnology in medicine has garnered significant interest, particularly in the development of advanced drug delivery systems. Graphene oxide (GO) shows promise as a carrier for delivering microRNA (miRNA) mimics or antisense constructs. miRNAs play a crucial role in regulating gene expression, and their dysregulation is associated with various diseases, including cancer. This study aimed to evaluate the impact of graphene oxide on cellular signaling pathways and its potential as a platform for gene delivery by developing a GO–antisense miRNA-21 nanosystem in HepG2 liver cancer cells. A colloidal dispersion of GO was used to prepare GO-antisense miRNA-21 nanosystems via self-assembly. The nanosystem was characterized in terms of ultrastructure, size distribution, surface composition and binding by TEM, DLS, ATR-FTIR and UV-Vis spectra. Zeta potential measurements were conducted to evaluate nanosystem stability by assessing the release kinetics of antisense miRNA-21. The efficiency of the GO nanosystem in delivering antisense miRNA-21 into HepG2 cells was analyzed using confocal microscopy and flow cytometry. Given the central role of miRNA-21 in inflammatory and oncogenic pathways, we first assessed its expression following GO exposure. In line with previous studies reporting high miRNA-21 expression in hepatocellular carcinoma cells, GO treatment further increased miRNA-21 levels in HepG2 cells compared with untreated controls. Changes in the expression levels of IL-8, MCP-1, ICAM-1, TIMP-2, and NF-kB were quantified by qPCR analysis. The ultrastructural analysis confirmed a strong affinity between GO and antisense miRNA-21. Transfection results demonstrate that the GO-based nanosystem effectively delivered antisense miRNA-21 into HepG2 cells, leading to a reduction in the expression of key pro-inflammatory genes. These findings suggest that GO-based nanocarriers may offer a promising strategy for delivering localized intratumoral miRNA-based therapies that target gene regulation in hepatocellular carcinoma.

## 1. Introduction

Graphene oxide (GO) is a remarkable two-dimensional nanomaterial derived from the oxidation of graphite [1]. This process introduces various oxygen-containing functional groups, such as hydroxyl, carboxyl, and epoxy, resulting in a layered, hydrophilic structure. Unlike pristine graphene, which is hydrophobic, GO disperses well in aqueous solutions. These functional groups not only enhance dispersibility but also provide a versatile platform for further chemical modifications. In addition, the high specific surface area, excellent loading capacity, and ease of functionalization make GO particularly well-suited for biomedical applications [2,3]. Although GO maintains mechanical strength and thermal stability similar to pristine graphene, the presence of oxygen functional groups disrupts the delocalized π-electron system, leading to a significant decrease in electrical conductivity [4]. This combination of characteristics makes GO particularly attractive for applications in drug delivery, bioimaging, and gene therapy [5,6,7]. One emerging application of GO is as a carrier for nucleic acids like microRNAs (miRNAs) [8,9,10]. miRNAs are small, non-coding RNA molecules that regulate gene expression at the post-transcriptional level [11,12]. They play vital roles in processes such as cell proliferation, differentiation, and apoptosis [13,14]. Dysregulation of miRNAs is associated with various diseases [15], including cancer [16], neurodegenerative conditions, and cardiovascular disorders [17]. Due to their therapeutic potential, the efficient and targeted delivery of miRNAs is essential for achieving clinical success. However, challenges remain, including their instability in physiological environments, susceptibility to enzymatic degradation, poor cellular uptake, and the need for precise control over their release [18,19]. To address these challenges, antisense miRNAs, engineered RNA sequences complementary to specific miRNAs, can be used to inhibit overexpressed miRNAs in cancer cells [20,21,22,23,24]. This suppression, which restores regular gene expression, offers a promising approach in cancer therapy [25]. Antisense miRNAs function by binding to target miRNAs, preventing them from interacting with their mRNA targets and blocking downstream effects. GO is a highly effective platform for delivering miRNAs due to its strong affinity for nucleic acids, facilitated by π-π stacking, electrostatic interactions and hydrogen bonding [6,26,27,28,29]. Additionally, functionalizing GO with biocompatible molecules improves its stability, reduces toxicity, and enhances cellular uptake, further increasing its value as a delivery vehicle [30]. This study investigates the application of GO as a delivery platform for antisense miRNA-21 in hepatocellular carcinoma cells, where miRNA-21 is frequently overexpressed and contributes to tumor progression by inhibiting tumor suppressor genes, enhancing proliferation, reducing apoptosis, and promoting metastasis and chemoresistance with a focus on evaluating its biological effects and gene-silencing efficacy. By leveraging GO’s unique properties, researchers aim to develop innovative gene regulation strategies with the potential to treat a wide range of diseases, particularly those linked to abnormal gene expression [31]. Ultimately, GO-based antisense miRNA delivery systems could represent a transformative advance in the treatment of genetic disorders and cancer.

## 2. Results

### 2.1. Characterization of Nanosystem

#### 2.1.1. Transmission, Scanning Transmission Electron Microscopy and Fourier Transform Infrared (FTIR) Spectrum

**Figure 1:**
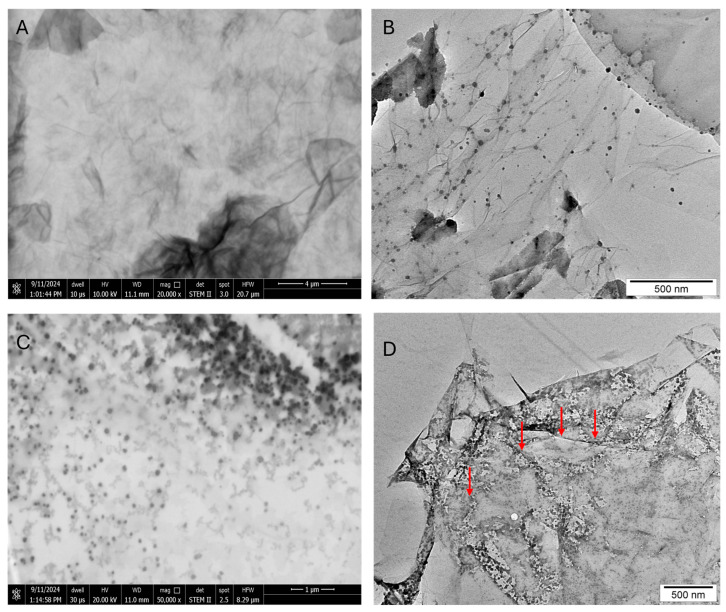
Ultrastructural evaluation of graphene oxide, antisense miRNA-21, and GO–antisense miRNA-21 nanosystem. Ultrastructure of graphene oxide (**A**,**B**), antisense miRNA-21 (**C**), GO–antisense miRNA21 nanosystems (**D**) analysed by transmission electron microscopy (**B**,**D**) and scanning transmission electron microscopy (**A**,**C**). Red arrows in panel (**D**) highlight localized interactions between GO and antisense miRNA-21 molecules, confirming effective self-assembly without evidence of aggregation. Scale bars: 500 nm–1 µm.

**Figure 2:**
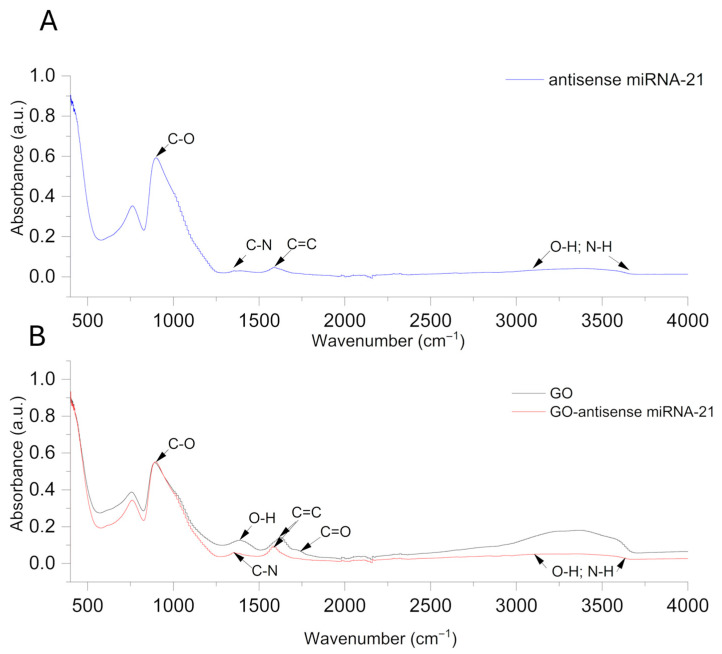
FTIR spectral analysis of GO, antisense miRNA-21, and GO–antisense miRNA-21 nanosystem. The attenuated total reflectance–Fourier transform infrared (ATR–FTIR) spectrum of antisense miRNA-21 (**A**), graphene oxide (**B**) and graphene oxide–antisense miRNA-21 nanosystem (**B**). GO at concentration 10.0 µg/mL, antisense miRNA-21 at 5 pmol and GO-antisense miRNA-21 nanosystems at concentration 10.0 µg/mL of GO and 5 pmol of antisense miRNA-21.

To gain insights into the structural organization of the GO–antisense miRNA-21 nanosystem and verify the successful self-assembly between GO and oligonucleotides, we performed ultrastructural and spectroscopic characterization. Transmission electron imaging techniques were employed to assess morphology, surface interactions, and nanosystem homogeneity, while FTIR spectroscopy facilitated the identification of characteristic functional groups and chemical bonding. The ultrastructure of graphene oxide (GO) (Figure 1A,B) after self-assembly with antisense miRNA-21 (Figure 1D) was investigated using transmission electron microscopy (Figure 1B,D) and scanning transmission electron microscopy (Figure 1A,C). Figure 1D (red arrows) revealed a strong affinity between antisense miRNA-21 and the flake surfaces of GO, without significant aggregation of either the miRNA sequences or GO sheets. This indicates that the self-assembly process is well-organized, and the antisense miRNA-21 binds efficiently to the GO without forming undesirable clusters. The FTIR spectrum of GO (Figure 2B) exhibited a prominent absorption band at 1725 cm^−1^, attributed to the stretching vibrations of C=O groups in carboxylic functionalities, and a band at 1620 cm^−1^, corresponding to the C=C skeletal vibrations of unoxidized graphitic domains. Additionally, the peaks observed around 1225 cm^−1^ and 1050 cm^−1^ are characteristic of C–OH and C–O–C stretching vibrations, respectively, confirming the presence of oxygen-containing groups on GO. Following functionalization with the antisense oligonucleotide, the FTIR spectrum of the GO–miRNA complex exhibited overall similar features to that of pristine GO, as expected given the relatively low oligonucleotide loading. However, a slight decrease in the broad O–H/N–H stretching region (3200–3500 cm^−1^) was observed, which may reflect the partial involvement of hydroxyl and amine groups in hydrogen bonding or electrostatic interactions with the oligonucleotide. Other spectral regions showed only minor or negligible variations, consistent with weak, non-covalent adsorption rather than covalent functionalization. For comparison, the spectrum of free miRNA (Figure 2A) showed the characteristic peaks of nucleobases and the phosphate backbone, including signals at 1650 cm^−1^ (amide I, mainly C=O stretching), 1535 cm^−1^ (amide II, N–H bending and C–N stretching), and a broad absorption near 1245 cm^−1^ corresponding to P=O stretching. Taken together, the comparison of pristine GO, free miRNA, and GO–miRNA complex spectra suggest the occurrence of a non-covalent association, mainly mediated by π–π stacking and hydrogen bonding, between the oligonucleotide and GO nanosheets.

#### 2.1.2. Dynamic Light Scattering, ζ-Potentials and UV-Vis

**Figure 3:**
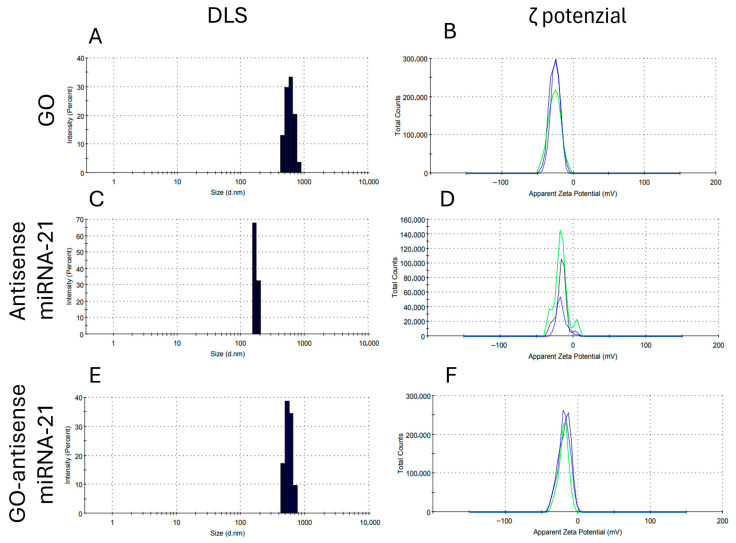
Particle size distribution and surface charge analysis of GO, antisense miRNA-21, and GO–antisense miRNA-21 nanosystem. Hydrodynamic diameter and ζ-potential values were measured for GO (**A**,**B**), antisense miRNA-21 (**C**,**D**), and the GO–antisense miRNA-21 nanosystem (**E**,**F**) using dynamic light scattering and electrophoretic mobility techniques. Measurements were performed in aqueous suspension at room temperature. Data are representative of at least n = 3 independent biological replicates. Colors in panels (**B**,**D**,**F**) represent data derived from independent biological replicates.

**Figure 4:**
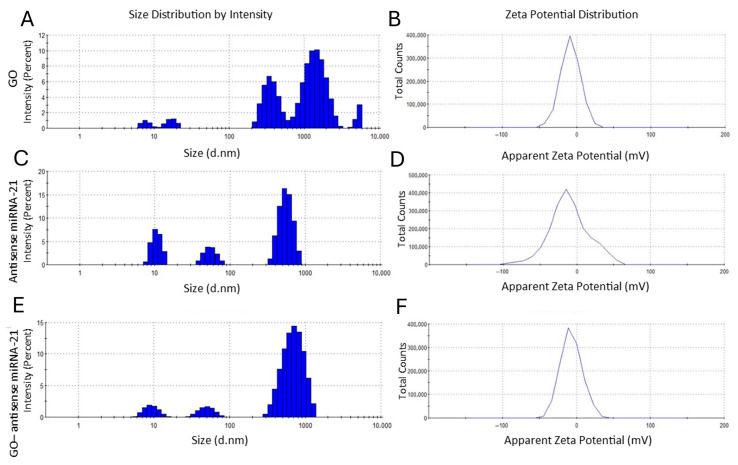
Particle size distribution and surface charge analysis of graphene oxide (GO), antisense miRNA-21, and the GO–antisense miRNA-21 nanosystem in serum-containing medium (10% FBS). Hydrodynamic diameter and ζ-potential values were determined for GO (**A**,**B**), antisense miRNA-21 (**C**,**D**), and the GO–antisense miRNA-21 nanosystem (**E**,**F**) using dynamic light scattering and electrophoretic mobility measurements. All analyses were performed in complete culture medium supplemented with 10% fetal bovine serum (FBS) at room temperature to assess colloidal behavior under biologically relevant conditions. Data are representative of at least three independent biological replicates (n ≥ 3).

In order to evaluate the physicochemical stability and colloidal behavior of the GO–antisense miRNA-21 nanosystem in aqueous solution, we measured particle size distribution and surface charge. These parameters are essential for predicting in vitro performance, cellular interaction, and systemic behavior. In parallel, UV–Vis spectroscopy was used to investigate optical properties and confirm nanosystem formation through characteristic absorbance signatures. The size distribution (Figure 3A,C,E) measured by DLS showed that GO exhibited a hydrodynamic diameter of approximately 800 nm (Figure 3A), while antisense miRNA-21 alone had a size of about 150 nm (Figure 3C). Upon complex formation, the GO-antisense miRNA-21 nanosystem showed an increased diameter pf approximately 950 nm (Figure 3E), indicating successful interaction and formation of a stable hydrocolloid. The ζ-potential (Figure 3B,D,F) reflects the surface charge and moderate stability of the nanosystems. ζ-potential of GO (Figure 3B), antisense miRNA-21 (Figure 3D) and GO–antisense miRNA-21 (Figure 3F) were −26.1 ± 7.43 mV, −16.2 ± 7.39 mV, and −18.7 ± 8.51 mV, respectively. The increase in ζ-potential upon antisense miRNA-21 loading (from −26.1 mV to −18.7 mV) suggests a partial neutralization of the GO surface charge, further supporting the successful formation of the GO–antisense miRNA-21 complex through electrostatic interactions. DLS and ζ-potential measurements were also performed in complete culture medium supplemented with fetal bovine serum (10% FBS) to assess the colloidal behavior of the GO–antisense miRNA-21 nanosystem under biologically relevant conditions (Figure 4). The size distribution profiles revealed a heterogeneous population, with a dominant fraction in the submicrometer range and additional smaller populations, indicating partial aggregation in the presence of serum proteins. Importantly, no rapid formation of large micron-scale aggregates was observed, suggesting that the system remains dispersed in serum-containing medium.

ζ-potential measurements in culture medium showed a shift toward less negative values compared to measurements performed in buffer, consistent with adsorption of serum proteins onto the GO surface and formation of a protein corona. Despite this shift, the zeta potential distribution remained relatively narrow, indicating a stable electrokinetic profile in biological medium.

**Figure 5:**
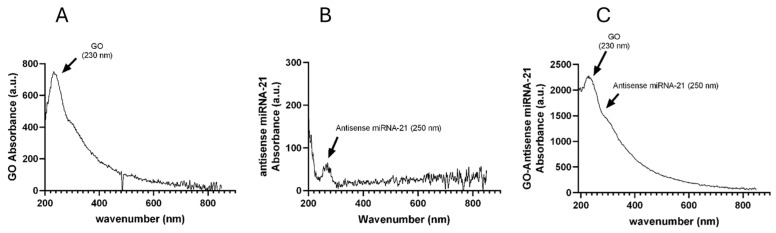
UV–Vis absorbance spectra of GO, antisense miRNA-21, and GO–antisense miRNA-21 nanosystem. Absorption spectra of GO (**A**), antisense miRNA-21 (**B**), and the GO–antisense miRNA-21 nanosystem (**C**) recorded over the 200–800 nm range. Data are representative of at least n = 3 independent biological replicates.

The UV-Vis spectrum analysis (Figure 5A–C) revealed some important features. The spectrum of pristine GO (Figure 5A) exhibited a strong absorption peak at approximately 230 nm, attributed to π–π* transitions of C=C bonds in the aromatic domains, and a shoulder around 300 nm, corresponding to n–π* transitions of C=O groups. The spectrum of antisense miRNA-21 (Figure 5B) was characterized by a prominent absorption peak in the 260–265 nm region, which is typical of nucleic acids and arises from π–π* transitions of the aromatic nucleobases. In the case of the GO–antisense miRNA-21 nanosystem (Figure 5C), the absorption profile appeared relatively broad and partially overlapping with the GO spectrum. Furthermore, a broadening and a variation in the intensity ratio between the absorption bands at approximately 250 and 230 nm was observed. Such a change may be indicative of alterations in the electronic structure of GO upon binding of the nucleic acid and suggest a potential interaction between GO and the antisense oligonucleotide. These observations are in line with the data obtained by FTIR, DLS, and zeta potential analyses, which collectively support the non-covalent binding of antisense oligonucleotides to the GO nanosheets.

#### 2.1.3. Results of Laser-Induced Breakdown Spectroscopy (LIBS)

**Figure 6:**
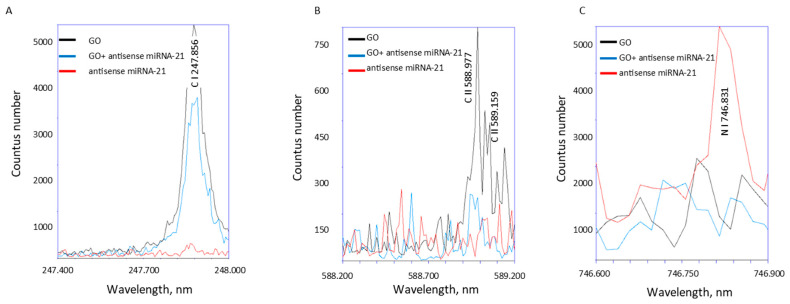
LIBS spectra of pristine GO and GO–antisense miRNA-21 nanosystem. The LIBS spectra of pristine GO and GO–antisense miRNA-21 nanosystem were recorded in the 200–900 nm range. The appearance of a nitrogen emission signal in the nanosystem spectrum, absent in pristine GO, confirms the presence of miRNA-21 sequences. Characteristic carbon emission lines (~247–248 nm) (**A**) indicate the preservation of sp^2^-hybridized carbon domains, while the decreased intensity of the C II line at 588.977 nm (**B**), and nitrogen N emission line at ~746.8 nm (**C**) in the functionalized sample suggests an effect of miRNA-21 binding on the ionization behavior of carbon species.

Laser-induced breakdown spectroscopy (LIBS) was employed to assess the elemental composition of graphene oxide and the GO-antisense miRNA-21 nanosystem. LIBS revealed a distinct nitrogen emission signal in the spectrum of the miRNA and GO-miRNA-21 (Figure 6), which was absent in GO. The presence of this signal indicates the successful conjugation of miRNA-21 to the GO surface, as nitrogen is a fundamental component of RNA nucleobases and is not present in unmodified GO. Furthermore, characteristic carbon (C) emission lines were observed in the ~247–248 nm (Figure 6A) region corresponding to sp^2^-hybridized carbon domains typical of the GO structure. These results confirm the preservation of the material’s structural integrity following functionalization. Additionally, the C II emission line at 588.977 nm, marked in the spectra (Figure 6B), was most intense in the GO sample (black line). The LIBS spectra revealed a distinct nitrogen (N) emission line at ~746.8 nm (Figure 6C) in the samples containing miRNA (pure miRNA and GO–miRNA-21 complex), which was absent in the spectrum of unmodified GO (Figure 6C). The presence of this nitrogen signal confirms the successful conjugation of miRNA-21 to the GO surface, as nitrogen is a major elemental constituent of RNA nucleobases and is not naturally present in GO. The reduced intensity of this peak of N in the GO-miRNA-21 (Figure 6C) conjugate indicates that miRNA functionalization may influence the ionization behaviour of carbon species on the GO surface.

The elemental composition inferred from LIBS was further corroborated by FTIR-ATR spectroscopy. The GO-miRNA-21 exhibited additional absorbance bands in the 1500–1700 cm^−1^ region (Figure 2), which can be attributed to N–H bending and amide vibrations. These spectral features support the presence of molecular interactions between nitrogen-containing particles of miRNA and the GO surface, providing further evidence of successful functionalization.

#### 2.1.4. Antisense miRNA-21 Release Kinetics from GO-Antisense miRNA-21 Nanosystem

In Vivo, the overexpression of miRNA-21 has been linked to various cancers and fibrotic diseases, making it a critical target for gene-silencing therapies. The efficacy of antisense oligonucleotides to inhibit miRNA-21 largely depends on the development of stable, biocompatible, and controlled delivery systems. Therefore, it is crucial to understand and optimize their behavior in the biological microenvironment. In this contest, we investigated the capability of a GO-based nanosystem to deliver antisense miRNA-21 by analyzing its release kinetics under physiologically relevant conditions. The release kinetics of antisense miRNA-21 from the GO-based nanosystem were evaluated at pH 4.5 and 7.4 to simulate physiologically and pathophysiologically relevant environments. The neutral pH 7.4 corresponds to the extracellular environment of healthy tissues and blood plasma, pH 4.5 reflects the late endosomal/lysosomal compartments, where nanocarriers are typically degraded or activated. Therefore, testing the nanosystem’s release at these specific pH values enables us to predict its behavior in both circulation and intracellular compartments, a crucial factor for achieving therapeutic efficacy.

**Figure 7:**
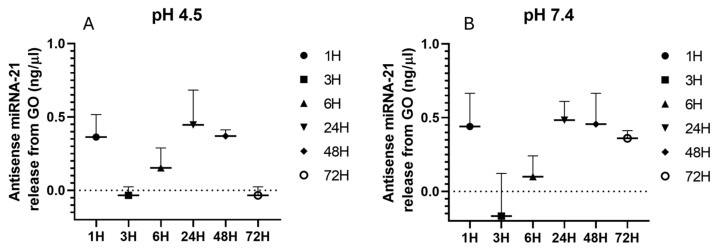
The kinetics of the release of antisense miRNA-21 from GO nanosystem were analyzed at two different pH levels 4.5 (**A**) and 7.4 (**B**) and at varying incubation times (1, 3, 6, 24, 48, and 72 h). The nanosystem was deposited as a dried nanofilm and incubated in PBS (pH 4.5 or 7.4) at 37 °C under rotary shaking (300 rpm). At predefined time points (1–72 h), samples were collected to quantify released nucleic acid. All measurements were performed on 100 µL aliquots and corrected for dilution. Data are representative of at least n = 3 independent biological replicates. The dashed line represents the baseline (blank) corresponding to the background signal in the measurement.

The results showed that at pH 4.5 the concentration of antisense miRNA-21 increased monotonically with increasing incubation time (Figure 7A). In contrast, at pH 7.4 the concentration peaked after 24 h before gradually decreased (Figure 7B). This enhanced release of antisense miRNA-21 at acidic pH may be attributed to the tumor-like microenvironment, which can alter the stability of the GO-antisense miRNA-21 complex through changes in surface charge, protonation of functional groups, and activation of biochemical mechanisms. The kinetic release trend demonstrates that release is enhanced under acidic conditions (pH 4.5) compared to physiological pH (7.4), which is consistent with the expected behavior of GO–miRNA systems due to protonation-induced weakening of electrostatic interactions.

To characterize the release mechanism, normalized release profiles (Mt/M∞) were fitted using the Higuchi, Korsmeyer–Peppas and Weibull models (Table 1). The Higuchi model exhibited high goodness-of-fit (R^2^ > 0.93), with higher k_H values at pH 4.5, indicating faster diffusion in acidic medium. The Korsmeyer–Peppas model produced diffusion exponents n = 0.32–0.41, consistent with Fickian diffusion. The Weibull model provided the best overall fit (R^2^ = 0.96–0.99), and β values (0.55–0.75) suggested diffusion through heterogeneous, multilayer GO structures. To complement the model-based analysis, descriptive kinetic parameters were calculated to provide an additional quantitative comparison between the two pH conditions. Descriptive metrics further supported accelerated release at acidic pH. The initial release rate (0–3 h) was approximately twice as high at pH 4.5. t_50_ values were 30–45% shorter at pH 4.5 than at pH 7.4. t_90_ was achieved within 48–60 h at pH 4.5, whereas it was not reached at pH 7.4. The 72 h release fraction remained substantially higher in acidic conditions. Together, these findings demonstrate that antisense miRNA-21 release from GO nanofilms is significantly faster and more complete at acidic pH, in agreement with the protonation-driven weakening of GO–RNA electrostatic interactions. This confirms the intended pH-responsive behavior of the nanosystem, supporting its suitability for intracellular delivery, where endosomal acidity is expected to trigger miRNA release.

These findings highlight the potential of GO-based carriers for targeted and pH-responsive delivery of therapeutic miRNAs, particularly in acidic tumor environments.

### 2.2. Cell Treatment

#### 2.2.1. Cellular Viability upon Nanosystem Treatment

**Figure 8:**
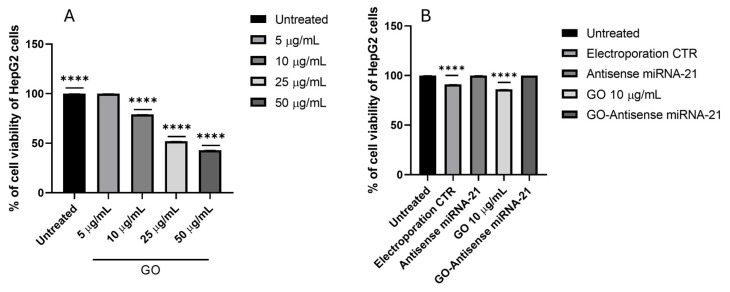
Cell viability of HepG2 cells after treatment with graphene oxide, antisense miRNA-21 and GO–antisense miRNA-21 nanosystems. (**A**) Viability (%) after 24 h exposure to increasing concentrations of GO (5–50 µg/mL), measured by MTT assay, untreated cells were used as control. (**B**) Analysis of cell viability after 24 h following treatment with GO (10 µg/mL), antisense miRNA-21 (5 pmol/mL), and GO–antisense miRNA-21 (10 µg/mL GO + 5 pmol/mL antisense). Data are expressed as percentage relative to untreated control cells. Data are presented as mean ± SD (n = 3). Statistical analysis was performed using one-way ANOVA followed by Tukey’s post hoc test. Asterisks indicate statistical significance: **** *p* < 0.0001. Horizontal lines above bars indicate the groups being compared. Abbreviation: Electroporation CTR: electroporation control.

Given that miRNA-21 is frequently overexpressed in hepatocellular carcinoma and plays a key role in promoting tumor progression, proliferation, and resistance to apoptosis, the HepG2 cell line was used as a relevant biological model to assess the effectiveness of antisense miRNA-21 delivery mediated by GO. Therefore, to ensure that the nanocarrier system is not intrinsically harmful to the cells, we evaluate cell citotoxicity after treatment with GO and the GO–antisense miRNA-21 nanosystem on HepG2 cells. The cells were treated with increasing concentrations of GO (5, 10, 25 and 50 µg/mL) for 24 h to assess dose-dependent cytotoxicity (Figure 8A). The results indicated a significant reduction in viability only at the highest concentrations tested, while GO up to 10 µg/mL showed moderate cytotoxicity, confirming its suitability for biological applications. The concentration of 10 µg/mL GO was selected based on previous studies that have reported its efficacy and low cytotoxicity in a variety of models [32,33]. Subsequently, the impact of GO-antisense miRNA-21 complex on cell viability was evaluated by treating HepG2 cells with GO (10 µg/mL), antisense miRNA-21 (5 pmol/mL, 0.036 ng/mL) by electroporation and nanosystem (GO 10 µg/mL; antisense miRNA-21 5 pmol/mL) for 24 h (Figure 8B), based on previous studies employing similar concentrations of antisense oligonucleotide [34]. Interestingly, although GO alone induced moderate cytotoxicity, we observed no significant reduction in cell viability with the GO–antisense miRNA-21 complex. This suggests that complexation of GO with antisense miRNA-21 may alter the surface properties of GO, potentially reducing the exposure of reactive groups responsible for cytotoxic effects, and thereby mitigating its toxicity. Our findings are consistent with several studies demonstrating that the surface chemistry and functionalization of GO are critical determinants of its biocompatibility and cellular uptake. These results suggest that the GO nanosystem is well tolerated by HepG2 cells at the concentrations used in this study, supporting its potential for safe and effective miRNA delivery in liver cancer models.

#### 2.2.2. Delivery Efficiency Evaluation of the GO-Antisense miRNA-21 Nanosystem

**Figure 9:**
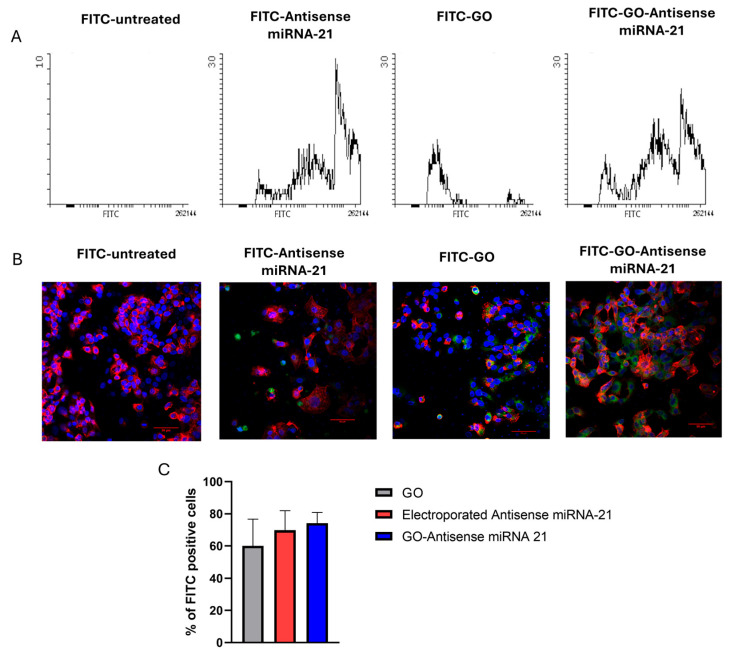
Evaluation of cellular uptake efficiency of GO, antisense miRNA-21, and GO–antisense miRNA-21 nanosystems in HepG2 cells. (**A**) Flow cytometry analysis of HepG2 cells treated with FITC-labeled GO, antisense miRNA-21, and GO–antisense miRNA-21 for 24 h. The x-axis represents FITC fluorescence intensity, corresponding to the intracellular uptake level of the labeled constructs. Fluorescence intensity was measured to determine the proportion of FITC-positive cells. (**B**) Confocal microscopy imaging of FITC-labeled (green color) constructs in HepG2 cells after 24 h incubation. Cells were counterstained with DAPI (nuclei- blue color) and ActinRed (cytoskeleton-red color). Scale bar: 50 µm. (**C**) Quantitative analysis by flow cytometry showing the percentage of FITC-positive cells after treatment with GO, antisense miRNA-21, and the GO-antisense complex. Data are presented as mean ± SEM.

The efficient delivery of therapeutic oligonucleotides into target cells remains a significant challenge in gene therapy. In this context, GO-based nanosystems offer promising delivery platforms due to their high surface area, biocompatibility, and ability to interact with nucleic acids and cellular membranes. To evaluate the cellular uptake and the delivery efficiency of the nanosystem, GO and antisense miRNA-21 were each conjugated with fluorescein isothiocyanate (FITC) via self-assembly. The delivery efficiency was first assessed by flow cytometry. The cytograms in Figure 9A show that FITC-conjugated GO, electroporated FITC-antisense miRNA-21, and FITC-GO–antisense miRNA-21 nanosystems effectively localized within HepG2 cells. In addition, the results demonstrated that the GO–antisense miRNA-21 construct exhibited higher transfection efficiency compared to electroporated-FITC-antisense miRNA-21. These findings support the potential of GO-based complexes as efficient carriers for gene delivery. Confocal microscopy analysis (Figure 9B,C) further confirmed these results, showing that a significantly higher number of FITC-positive HepG2 cells following treatment with the FITC-GO–antisense miRNA-21 nanosystem compared to treatment with either FITC-conjugated GO or electroporated FITC-antisense miRNA-21.

#### 2.2.3. Gene Expression Modulation by GO and GO–Antisense miRNA-21 Nanosystem

**Figure 10:**
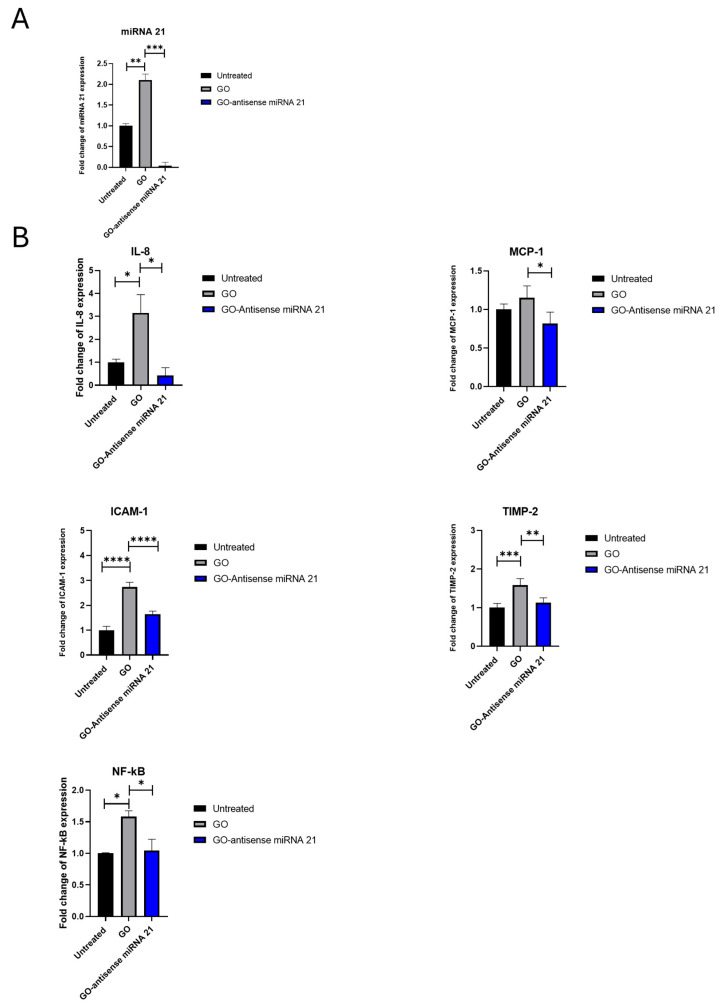
Quantitative real-time PCR analysis of miRNA expression and pro-inflammatory and tumor-related gene expression in HepG2 cells. (**A**) Transcriptional levels of miRNA-21 in untreated HepG2 cells and in cells treated with GO (10 µg/mL) and with Antisense miRNA-21 (5 pmol/mL) for 24 h. (**B**) Transcriptional levels of IL-8, MCP-1, ICAM-1, TIMP-2 and NF-κB in HepG2 cells. The cells were untreated and treated with GO (10 µg/mL) and GO-antisense miRNA-21 (GO 10 µg/mL + antisense miRNA-21 5 pmol/mL) for 24 h. After, the cells were collected for total RNA extraction. The transcriptional levels of the considered genes were analyzed by calculating the value of 2^−ΔΔCt^. The assay was performed in triplicate ± SD and expressed as a fold change over the housekeeping genes. **** *p* < 0.0001; *** *p*< 0.001; ** *p* < 0.01; * *p* < 0.05.

To further elucidate the biological effects of GO and its potential as a nanocarrier for therapeutic nucleic acids, the endogenous levels of miRNA-21 in HepG2 cells and their modulation following GO exposure were examined. This initial analysis was crucial for clarifying whether GO-induced modulation of miRNA-21 could act as an upstream regulator of the inflammatory and tumor-related genes examined in the subsequent experiment, and to evaluate whether the antisense-functionalized nanosystem could effectively counteract these effects. As shown in Figure 10A, HepG2 cells exhibited intrinsically high basal expression of miRNA-21, which is consistent with previous reports [35,36]. Furthermore, treatment with GO alone triggers cellular signaling events that culminate in the upregulation of miRNA-21. This observation suggests that GO can potentiate the upregulation of this oncomiR as part of a general stress-related cellular response. Conversely, treatment with GO–antisense miRNA-21 nanosystem resulted in a marked reduction in endogenous miRNA-21 levels, confirming the efficacy of the antisense construct in suppressing the target miRNA. We next evaluated the impact of GO and a GO-antisense miRNA-21 nanosystem on the gene expression profile of HepG2 cells (Figure 10B). Our research focused on a set of genes involved in inflammation and tumor progression such as interleukin 8 (IL-8), intercellular adhesion molecule 1 (ICAM-1), monocyte chemoattractant protein-1 (MCP-1), metallopeptidase inhibitor 2 (TIMP-2) and the transcription factor NF-kB. Among these targets, IL-8, MCP-1, and NF-κB have been previously identified as direct or indirect targets of miRNA-21-mediated regulation in various cancer models, including liver cancer [37,38,39,40]. In particular, miRNA-21 has been shown to modulate NF-κB signaling, contributing to a pro-inflammatory and pro-tumorigenic microenvironment. Similarly, MCP-1 and IL-8 expressions have been linked to miRNA-21-dependent mechanisms that enhance chemotactic signaling and inflammatory cell recruitment. In contrast, although ICAM-1 and TIMP-2 play relevant roles in tumor progression and extracellular matrix remodeling, the current literature does not provide direct evidence of their transcriptional regulation by miRNA-21. Their inclusion in our analysis aims to explore potential downstream effects of miRNA-21 inhibition or off-target influences of the GO-based delivery system, contributing to a broader understanding of gene network perturbations under these experimental conditions. Therefore, HepG2 cells were treated with GO (10 µg/mL) or with the GO–antisense miRNA-21 nanosystem. After 24 h, total RNA was extracted and analyzed using real-time PCR to quantify changes in gene expression. GO treatment was associated with an increased expression of the analyzed genes, including IL-8, ICAM-1, TIMP-2, and NF-κB, which may reflect a stress-related cellular response. No statistically significant increase was observed for the MCP-1 gene expression. Conversely, cells treated with the GO–antisense miRNA-21 nanosystem showed reduced expression levels of these same genes compared to the GO-treated cells. Taken together, the miRNA-21 upregulation induced by GO (Figure 10A), combined with the concomitant upregulation of inflammatory and tumour-related genes (Figure 10B), and their subsequent downregulation by the GO–antisense miRNA-21 nanosystem, strongly supports the conclusion that the observed transcriptional repression is specifically attributable to antisense-mediated inhibition of miRNA-21 delivery.

## 3. Discussion

Hepatocellular carcinoma (HCC) is one of the most prevalent and lethal malignancies worldwide, with a high mortality rate due to late diagnosis and limited therapeutic efficacy of current treatments [41,42]. Standard clinical approaches include surgical resection, liver transplantation, chemotherapy, and targeted therapies. However, these are often insufficient, particularly in advanced-stage HCC, underscoring the urgent need for innovative therapeutic strategies. One such approach involves the use of molecules capable of modulating gene expression, such as antisense oligonucleotides (ASOs). Among the targets of interest, microRNAs (miRNAs) have attracted increasing attention due to their regulatory roles in key biological processes, including cell proliferation, apoptosis, and metastasis. miRNA-21 is one of the most studied oncogenic miRNAs, frequently overexpressed in HCC. It contributes to tumor progression, drug resistance, and fibrogenesis, making it an attractive therapeutic target [43]. Antisense oligonucleotides against miRNA-21 can bind selectively to the mature miRNA, preventing it from interacting with its mRNA targets and thereby restoring normal gene expression profiles [44]. In this context, graphene oxide (GO) has emerged as a promising vector for antisense miRNA delivery. Thanks to its high surface area, π–π stacking capacity, and ease of functionalization, GO provides a stable and biocompatible platform for nucleic acid loading and targeted release. Additionally, its two-dimensional structure enhances cellular uptake and protects miRNAs from enzymatic degradation [45,46,47]. These properties make GO-based delivery systems particularly appealing for gene therapy in cancer. In this study, we developed and characterized a GO–antisense miRNA-21 nanosystem and investigated its biological effects on HepG2 cells. Our results demonstrate that the nanosystem exhibits moderate stability under experimental conditions, efficient loading of the antisense oligonucleotide, and a pH-responsive release behavior that favors acidic tumor-like environments. Furthermore, the average hydrodynamic size of the complex, as determined by DLS, was higher than that observed by direct imaging techniques such as TEM. This expected discrepancy can be attributed to the contribution of the solvation shell, electrostatic surface interactions, and potential soft agglomeration in aqueous solution. Comparison of the colloidal properties of the GO–antisense miRNA-21 nanosystem in buffer and in serum-containing culture medium highlights the strong influence of biological components on its physicochemical behavior. While measurements performed in serum-free conditions showed relatively narrow size distributions typical of dispersed GO sheets, incubation in complete medium supplemented with FBS resulted in broader, more heterogeneous size profiles. This behavior is consistent with the adsorption of serum proteins onto the GO surface and the formation of a protein corona [48]. Importantly, despite these changes, no extensive aggregation into large micrometer-scale structures was observed, indicating that the nanosystem remains dispersed under biologically relevant conditions. The concomitant shift in the zeta potential toward less negative values further supports protein adsorption and electrostatic screening rather than loss of colloidal integrity. Together, these findings suggest that the GO–antisense miRNA-21 nanosystem exhibits moderate colloidal stability in serum-containing environments, which is sufficient for preliminary in vitro biological studies and supports its use as a platform for investigating intracellular delivery mechanisms rather than immediate systemic application. The combined use of DLS and TEM provides a more comprehensive understanding of nanoparticle behavior in both hydrated and dehydrated states, in line with international recommendations for nanomaterial characterization. These include the European Commission Recommendation 2011/696/EU on the definition of nanomaterials, the OECD Guidance Document No. 125, and the EFSA Scientific Committee guidelines (2021) [49]. Although characterization in biologically relevant media, such as cell culture medium or serum-supplemented DMEM, can provide useful information about nanoparticle behavior under physiological conditions, these media introduce significant analytical challenges. The complex composition, including high ionic strength, variable viscosity, abundant proteins, affects particle mobility and scattering, may result in soft agglomeration or dynamic protein corona formation. These factors can unpredictably influence both hydrodynamic size and zeta potential, leading to inconsistent and non-reproducible measurements. In particular, zeta potential determinations based on electrophoretic mobility (e.g., using the Smoluchowski approximation) assume constant viscosity and dielectric properties, which are not met in culture media. As acknowledged in ISO/TR 13014:2012 [50] and OECD Guidance Document No. 317 [51], measurements in aqueous systems (e.g., deionized water or PBS) remain the gold standard for inter-study comparability and methodological reproducibility.

It should be emphasized that the GO–antisense miRNA-21 nanosystem investigated in this study is not primarily intended for systemic intravenous administration. Instead, its proposed mode of application is direct intratumoral injection, a clinically established approach used for the treatment of solid, non-resectable tumors. Local administration allows for high local concentrations of the therapeutic agent at the tumor site while minimizing systemic exposure, thereby reducing concerns related to circulation stability, serum-induced aggregation, and off-target effects.

Complementary spectroscopic analyses further supported the structural integration of the antisense oligonucleotide with GO. UV–Vis and LIBS measurements confirmed the presence of antisense miRNA-21 sequences not only adsorbed on the surface of the graphene oxide flakes but also distributed within inter-flake regions. Notably, LIBS analysis revealed a uniform nitrogen signal throughout the entire sample, indicative of a high degree of nanosystem functionalization. The spatially widespread detection of nitrogen suggests that antisense miRNA-21 was incorporated not only onto the external surfaces but also into interlayer domains, demonstrating the deep and homogeneous modification of the GO nanostructure. Moreover, the obtained release kinetics results are consistent with previous reports, which have shown that GO-based matrices exhibit diffusion-controlled release due to their lamellar architecture, variable interlayer hydration, and heterogeneous porosity [52]. The pH-dependent behavior reflects protonation of GO functional groups at low pH, leading to reduced electrostatic attraction and accelerated oligonucleotide desorption, a mechanism similarly described for GO–DNA and GO–siRNA conjugates [53]. Importantly, the films retained miRNA efficiently at physiological pH while allowing for rapid release under acidic conditions that mimic endosomal, lysosomal, and finally intratumoral environments. This dual behavior is desirable for intracellular antisense delivery, as it provides stability during circulation and efficient release upon cellular internalization. Comparable pH-triggered responses have been documented in GO-based nucleic acid delivery systems, highlighting protonation-mediated weakening of π–π stacking and hydrogen bonding at acidic pH, which facilitates intracellular unloading [53]. The cytotoxicity data demonstrated that GO at a concentration of 10 µg/mL caused a moderate reduction in HepG2 cell viability (~25%), whereas the GO–antisense miRNA-21 complex induced a less pronounced decrease. The cytotoxic effects observed for the GO–antisense miRNA-21 nanosystem should therefore be interpreted in the context of both dose and cellular background. Graphene oxide is known to induce oxidative stress and membrane perturbation in cancer cells at higher concentrations [9,26,33]. Notably, in the present study, the GO–antisense miRNA-21 complex did not merely induce nonspecific cytotoxicity but was associated with a coordinated downregulation of oncogenic and pro-inflammatory pathways linked to miRNA-21 signaling. This suggests that the observed reduction in cell viability reflects, at least in part, a biologically relevant therapeutic response rather than purely material-induced toxicity.

Moreover, the proposed mode of application for this nanosystem is local intratumoral administration, where controlled, site-specific exposure is expected to mitigate systemic toxicity. Collectively, these findings suggest that conjugation of antisense miRNA-21 to GO may partially mitigate the inherent cytotoxicity of GO, possibly by altering its physicochemical interactions with cellular membranes. This interpretation is supported by previous studies demonstrating that the surface chemistry and functionalization of GO strongly influence its biocompatibility and cellular uptake [54,55,56,57,58].

In this context, complexation with antisense miRNA-21 may reduce the availability of oxygen-containing functional groups and sharp edges of GO that are otherwise associated with oxidative stress or membrane disruption. Confocal microscopy and flow cytometry demonstrated markedly enhanced cellular uptake of the GO–antisense miRNA-21 nanosystem compared with free miRNA, confirming the role of GO in promoting internalization. Flow cytometric analysis using FITC-labeled constructs revealed a clear fluorescence shift across the treated cell population, providing quantitative evidence of effective intracellular uptake. Although confocal microscopy and flow cytometry demonstrated efficient cellular uptake of the GO–antisense miRNA-21 nanosystem, direct subcellular colocalization analyses were not performed in this study. Therefore, precise intracellular compart-mentalization cannot be conclusively established. Nevertheless, the robust downregulation of miRNA-21 and its downstream target genes provides strong functional evidence that the antisense oligonucleotide was effectively delivered into the intracellular environment, where it could exert its biological activity. Recognizing the central role of this oncomiR in inflammatory, pro-survival, and adhesion-related pathways in hepatocellular carcinoma (HCC), it was essential to determine whether GO itself could modulate miRNA-21 expression prior to analyzing the downstream transcriptional responses. Although miRNA-21 expression is commonly reported as intrinsically elevated in HCC cells, our data reveal that GO exposure induced an additional upregulation of miRNA-21 levels in HepG2 cells compared with untreated controls. This data provides a mechanistic basis for the subsequent activation of inflammation- and tumor-associated genes observed in our study, strongly supporting the notion that miRNA-21 may function as an upstream mediator of the cellular stress response to GO. Real-time PCR analysis supported this interpretation. Indeed, GO treatment resulted in the upregulation of several miRNA-21 responsive genes involved in inflammation and tumor progression including *IL-8*, *ICAM-1*, *TIMP-2*, and *NF-κB*. This effect may be attributed to a stress response to nanomaterial, possibly involving the activation of pro-inflammatory and adhesion-related signaling pathways, as has been observed in previous studies using GO-based materials [59,60,61]. Interestingly, when antisense miRNA-21 was delivered via GO, a clear downregulation of all these genes was observed compared to GO alone. This result confirms the efficacy of the GO nanosystem in silencing miRNA-21 and modulating downstream gene expression. The suppression of *IL-8* and *MCP-1* suggests a potential anti-inflammatory effect, as these cytokines are involved in leukocyte recruitment and tumor-promoting inflammation [62,63]. *ICAM-1* downregulation may reflect a reduced adhesion potential, which could impair tumor cell migration and metastatic spread. The observed decrease in *NF-κB* expression is particularly relevant, since this transcription factor is a central regulator of many pro-inflammatory and pro-survival genes, including those mentioned above. Therefore, the reduction in *NF-κB* could contribute broadly to the transcriptional repression seen in this study. Among the most interesting findings is the decrease in *TIMP-2* expression following treatment with the GO–antisense miRNA-21 complex. *TIMP-2* is known not only for its role as an inhibitor of matrix metalloproteinases but also as a key player in liver fibrosis and tumor-stroma interactions [64,65,66,67]. The observed reduction may therefore indicate not only an anti-tumoral response but also a potential anti-fibrotic effect, which is of particular importance in the context of HCC, where fibrotic remodeling of the microenvironment plays a critical role in disease progression and therapeutic resistance. Several studies have reported that the interaction between graphene oxide and nucleic acids, primarily through π–π stacking and hydrogen bonding, may enable its use as an effective nanocarrier for gene delivery applications. In line with this, our findings indicate that GO can facilitate the intracellular delivery of antisense miRNA-21 in hepatocellular carcinoma cells. While our in vitro results provide strong evidence of its biological activity, further in vivo studies are necessary to fully assess its therapeutic potential.

## 4. Materials and Methods

### 4.1. Nanosystem

#### 4.1.1. Graphene Oxide

Graphene oxide dispersion (purity 99.99%) was purchased from Graphenea (San Sebastián, Spain). Antisense miRNA-21 (UAGCUUAUCAGACUGAUGUUGA; HSTUD0399) was obtained from [Sigma-Aldrich, St. Louis, MO, USA]. All reagents used were of analytical grade. Cell culture reagents, including Dulbecco’s Modified Eagle Medium (DMEM), fetal bovine serum (FBS), and antibiotics, were supplied by Sigma-Aldrich (St. Louis, MO, USA) unless otherwise specified.

#### 4.1.2. Synthesis of Graphene Oxide–Antisense miRNA-21 Nanosystem

GO was diluted to a working concentration of 10 µg/mL in sterile ultrapure water and sonicated for 30 min (40 kHz; Vibra-Cell ultrasonic processor, Sonics & Materials Inc., Newtown, CT, USA) to ensure uniform dispersion. Antisense miRNA-21 was prepared at a final concentration of 5 pmol/mL and mixed with the GO suspension. The mixture was further sonicated for 30 min to promote self-assembly. The nanosystem was incubated overnight at 4 °C under constant agitation (200 rpm) before use in subsequent experiments.

### 4.2. Characterization of the Nanosystem

#### 4.2.1. Transmission Electron Microscopy

The shape and size of the GO, antisense miRNA-21, and GO-Antisense miRNA-21 were inspected using a transmission electron microscope (TEM; TEM.JEOL JEM-1220, JEOL Ltd., Tokyo, Japan). The morphology of GO and GO-Antisense miRNA-21 was inspected using a transmission electron microscopy (TEM.JEOL JEM-1220, JEOL Ltd., Tokyo, Japan) at 80 KeV equipped with an 11-megapixel camera (Morada TEM, Olympus Corporation, Tokyo, Japan). Triplicate samples of GO and GO-antisense miRNA-21 were prepared for TEM by placing droplets of the hydrocolloid onto Formvar-coated copper grids (Agar Scientific Ltd., Stansted, UK) and air drying before TEM imaging.

#### 4.2.2. Fourier Transform Infrared (FTIR) Spectroscopy

The FTIR spectra of dried samples were recorded using an FTIR spectroscope with a diamond ATR pickup (Nicolet 6700, Thermo Scientific, Waltham, MA, USA). All samples were prepared by dropping 200 µL of sample suspension on a microscope glass and drying the suspension at 40 °C. Each spectrum was rationed to a background spectrum previously registered with an empty measuring chamber. This was performed to remove the effect of carbon dioxide and water vapour present in the laboratory air.

#### 4.2.3. Dynamic Light Scattering and ζ-Potentials

Particle sizes were measured in a particle size analyser (Zetasizer ZSP, Malvern Instrument Ltd., Worcestershire, UK) at 25 °C based on laser Doppler velocimetry and dynamic light scattering (DLS) techniques. Previously, the suspension was homogenised using an ultrasonication probe for 30 min. The ζ-potentials of GO, antisense miRNA-21, and GO-antisense miRNA-21 were measured by the dynamic laser scattering electrophoretic method, using the Smoluchowski approximation with a zeta potential analyser (Zetasizer Nano ZS90 Malvern Instruments, Malvern, UK) in aqueous suspension and in complete cell culture medium supplemented with 10% fetal bovine serum (FBS) to evaluate colloidal behavior under biologically relevant conditions. Each sample was measured after stabilisation at 25 °C for 120 s. All measurements were performed in triplicate.

#### 4.2.4. UV-Visible Spectroscopy

UV-Vis spectra were recorded using a Nanodrop One (Thermo Fisher Scientific, Waltham, MA, USA) in the range of 200–800 nm. Characteristic absorption peaks confirmed the interaction between miRNA and GO.

#### 4.2.5. Release Kinetics of Antisense miRNA-21

PBS solutions at pH 7.4 and 4.5 were separately introduced into a GO-Antisense miRNA-21 nanofilm that had been air-dried under sterile conditions. The samples were incubated at 37 °C under continuous agitation at 300 rpm. The concentration of antisense miRNA-21 released from GO-antisense miRNA-21 nanosystem was quantified using a Nanodrop One spectrophotometer by measuring absorbance at (Thermo Fisher Scientific, Waltham, MA, USA) at 260 nm. For each measurement, 100 μL of PBS (pH 7.4 or pH 4.5) was used. Following sample collection, an equivalent volume of fresh PBS at the corresponding pH was added to maintain a constant total volume. The samples were then subsequently returned to rotary shaking until the next sampling point. The samples were subsequently returned to rotary shaking until the next sampling point. Measurements were performed after incubation periods of 1, 3, 6, 24, 48, and 72 h, and the cumulative release percentage of antisense miRNA-21 was calculated and plotted as a function of time.

To characterize the release mechanism, the normalized cumulative release profiles (*M_t_*/*M*_∞_) using three kinetic models: the Higuchi model, the Korsmeyer–Peppas model (applied within the *M_t_*/*M*_∞_ ≤ 0.6 region), and the empirical Weibull model, were analyzed. These models were selected because they are well established for describing diffusion-driven release from thin polymeric or layered matrices. The Higuchi model enables the approximation of release under Fickian diffusion from planar structures, while the Korsmeyer–Peppas model facilitates the mechanistic classification of the release process through its diffusion exponent. The Weibull model was included due to its flexibility and suitability for heterogeneous materials, providing robust characterization of release profiles that may not follow idealized diffusion behavior. Together, these models offer complementary perspectives for evaluating release kinetics from GO nanofilms under different pH conditions. To ensure precise and comparable kinetic modeling, all raw absorbance measurements were systematically preprocessed and converted into normalized fractional release values (*M_t_*/*M*_∞_). The resulting standardized dataset formed the foundation for both descriptive kinetic analysis and model fitting.

Data preprocessing and normalization- absorbance values were processed in three steps:

1. Baseline correction
  For each release condition, the minimum absorbance value (*A*_min_) within the dataset was subtracted from all measurements:
  $$A_{\mathrm{corr}}(t_{i})=A(t_{i})-A_{min}$$
2. Normalization to maximum corrected absorbance
  The corrected absorbance was divided by the maximum corrected value (*A*_corr,max_) for that condition:
  $$M_{t}/M_{\infty }(t_{i})=\frac{A_{\mathrm{corr}}(t_{i})}{A_{\mathrm{corr},\max}}$$

This procedure yields dimensionless fractional release profiles (0–1) independent of batch variability.

3. Time alignment
  Time labels of 1 h, 3 h, 6 h, 24 h, 48 h and 72 h (and 168 h where applicable) were converted to numerical hours and paired with normalized release values.
  The final dataset comprised time, experimental condition, mean absorbance, and the ratio *M_t_*/*M*_∞_ for each sample. This dataset was utilized for both kinetic modeling and descriptive quantitative analysis. To characterize the dominant release mechanism, the normalized profiles (*M_t_*/*M*_∞_) were fitted using three established kinetic models:

Higuchi model (diffusion-controlled release):

$$M_{t}/M_{\infty }=k_{H}\;t^{1/2}$$

Korsmeyer–Peppas model-applied only within the initial linear region (*M_t_*/*M*_∞_ ≤ 0.6):

$$M_{t}/M_{\infty }=k_{KP}\;t^{n}$$

where the diffusion exponent *n* ≤ 0.45 is indicative of Fickian diffusion in thin films.

Weibull empirical model-suitable for structurally heterogeneous systems such as multilayered GO films:

$$M_{t}/M_{\infty }=1-exp[-{\left(\frac{t}{\tau }\right)}^{\beta }]$$

Model fitting was performed using nonlinear least-squares regression to estimate all kinetic parameters, and the quality of each fit was evaluated using the coefficient of determination (R^2^). The Korsmeyer–Peppas model was applied only when at least three data points were available within the *M_t_*/*M*_∞_ ≤ 0.6 region to ensure reliable parameter estimation. All three models were fitted independently for each experimental condition (pH 4.5, pH 7.4, and PBS).

#### 4.2.6. Laser-Induced Breakdown Spectroscopy (LIBS) Analysis

Laser-induced breakdown spectroscopy (LIBS) was performed to characterize the elemental composition of graphene oxide and GO–antisense miRNA-21 nanosystems. Nanoparticle suspensions were prepared from both formulations and drop-cast onto 2-inch monocrystalline silicon wafers (Dummy CZ-Si, Microchemicals, Ulm, Germany), followed by vacuum drying at 50 °C for 24 h. LIBS measurements were conducted using a Nd:YAG laser system (Quantel Brio, Les Ulis, France) operating at a fundamental wavelength of 1064 nm with a pulse duration of 4 ns, pulse energy of 46 mJ, and a gate delay of 500 ns. Emission from the induced plasma was collected via an optical fiber and analyzed using a broadband spectrometer (Avantes, Apeldoorn, The Netherlands). Spectral acquisition was conducted over the 200–900 nm range to detect potential emission lines corresponding to trace elements or functional groups. All spectra were background-corrected and normalized for comparative analysis.

### 4.3. Cell Cultures and Treatments

#### 4.3.1. Cell Line and Conditions

HepG2 human liver carcinoma cells (ATCC^®^ HB-8065™) were cultured in DMEM supplemented with 10% FBS and 1% penicillin/streptomycin. Cells were maintained at 37 °C in a humidified incubator with 5% CO_2_.

#### 4.3.2. Electroporation with Antisense miRNA-21

For transfection by electroporation, HepG2 cells were cultured in a 25 cm^3^ flask (3 × 10^6^ cells). After collection, the cells were suspended into 200 µL of electroporation buffer (165–2676, Bio-Rad, Hercules, CA, USA) for a final cell concentration of 1 × 10^6^ and placed in a 0.2 µm electroporation cuvette (165–2092, Bio-Rad). Next, 2 µL of antisense miRNA-21 were introduced into the HepG2 cells. Electroporation was conducted under the following conditions: voltage 200 V, capacitance 900 F, pulse duration 20 ms, resistance 20 and single pulse using Gene Pulser Xcell Electroporation Systems (Bio-Rad, Hercules, CA, USA).

#### 4.3.3. Treatment with GO Complex

For transfection, the cells were cultured in six-well plates (1 × 10^5^ cells per well) and the GO-antisense miRNA 21 (GO 10.0 µg/mL: miRNA 5.0 pmol/mL). After transfection, the cells were cultivated in six-well plates (1 × 10^5^ cells per well) for 24 h. After 24 h, the cells were collected for further studies.

#### 4.3.4. Cell Viability Assay

HepG2 cell viability was assessed using the XTT (2,3-bis(2-methoxy-4-nitro-5-sulfophenyl)-2H-tetrazolium-5-carboxyanilide) assay (Life Technologies, Taastrup, Denmark) after 24 h of incubation. This exposure time was selected following the recommendations of the ISO standard ISO 10993-5:2009 [68], which defines protocols for in vitro cytotoxicity testing of medical materials, including nanomaterials. Additionally, the procedure adhered to guidance from ISO/TR 10993-22:2017 [69], which addresses testing considerations specific to nanomaterials in biological systems [70]. For the cell viability assay, the selected cell lines were first seeded into 96-well plates at a density of 5 × 10^3^ cells per well. GO hydrocolloid suspensions were then administered at final concentrations of 5, 10, 25, and 50.0 µg/mL. After exposure to GO for 24 h, 100 µL of XTT reagent was added to each well. The plates were subsequently incubated at 37 °C for an additional 3 h to allow for colorimetric development prior to analysis. Next the optical density (OD) of each well was measured at 450 nm using spectrophotometer (Infinite M200, Tecan, Durham, NC, USA). Cell viability was expressed as a percentage (ODtest − ODblank)/(ODcontrol − ODblank), where “ODtest” is the OD of cells exposed to GO, “ODcontrol” is the OD of the control sample and “ODblank” is the OD of wells without cancer cells.

#### 4.3.5. Cell Viability Assay After Antisense miRNA-21 and GO-Antisense miRNA-21 Nanosystem Administration

The GO–antisense miRNA-21 nanosystem, consisting of graphene oxide (10.0 µg/mL) loaded with antisense miRNA-21 (5.0 pmol/mL), as well as free antisense miRNA-21, were evaluated for their effects on cell viability. The assay was performed using a 2,3-bis-(2-methoxy-4-nitro-5-sulfophenyl)-2H-tetrazolium-5-carboxanilide (XTT) cell viability kit (Life Technologies, Taastrup, Denmark) following 24 h of treatment, as previously described. HepG2 cells were seeded in 96-well plates at a density of 5 × 10^3^ cells per well. The cells were then treated with the GO–antisense miRNA-21 nanosystem (GO 10.0 µg/mL; miRNA 5.0 pmol/mL), GO alone (10.0 µg/mL), or antisense miRNA-21 alone (5.0 pmol/mL). Treatments were applied either directly to the culture medium or via electroporation, followed by a further 24 h incubation.

Subsequently, 100 µL of XTT reagent was added to each well, and the plates were incubated for an additional 3 h at 37 °C. Absorbance was recorded at 450 nm using a multiwell scanning spectrophotometer (Infinite M200, Tecan, Durham, NC, USA).

### 4.4. Delivery Efficiency

#### 4.4.1. FITC Labeling and Confocal Microscopy

GO, antisense miRNA-21, and their complexes were labeled with fluorescein isothiocyanate (FITC, Thermo Fisher Scientific, Waltham, MA, USA) at a concentration of 0.1 mg/mL and incubated overnight at 4 °C in the dark. HepG2 cells were then treated with FITC-labeled GO, antisense miRNA-21, and the corresponding nanosystem. After 24 h, cells were fixed with 4% paraformaldehyde and stained with DAPI and ActinRed. Confocal images were acquired using an IX81 FV1000 microscope (Olympus, Tokyo, Japan).

#### 4.4.2. Flow Cytometry

Delivery efficiency was assessed using a flow cytometer (FACSCalibur, Becton Dickinson, Franklin Lakes, NJ, USA). For analysis of FITC-positive HepG2 cells treated with GO–FITC, antisense miRNA-21–FITC, or GO–antisense miRNA-21–FITC, the cells were seeded in 6-well plates at a density of 1 × 10^6^ cells/mL and maintained under standard culture conditions for 24 h prior to treatment. After the incubation time, cells were treated for 24 h with the GO-FITC, Antisense miRNA-21-FITC and GO- Antisense miRNA-21-FITC. Next, cells were collected into the flow cytometry tubes and suspended in PBS. During the investigation the experiment was performed in triplite. Fluorescence emission intensity was measured using FL1 channels for FITC at Em = 530 nm using excitation at 488 nm. Histograms were generated using Flowing Software 2.5.1 (Perttu Terho, Turku, Finland).

### 4.5. Total RNA Extraction and Real-Time PCR

#### 4.5.1. RNA Extraction, cDNA Synthesis and Real Time-PCR

Total RNA extraction was performed using the PureLink^®^ RNA Mini Kit (Ambion™ Life Technologies, Foster City, CA, USA). Briefly, harvested cell pellets were resuspended in lysis buffer supplemented with 1% 2-mercaptoethanol and subsequently homogenized using a TissueLyser ball mill (Qiagen, Germantown, MD, USA) for two cycles of 5 min at a frequency of 50 Hz. The probes centrifuged at 12,000× *g*, and the pellets were discarded. The supernatant with total RNA was transferred into a new clean tube and underwent the manufacturer’s instructions. The RNA samples were eluted in 50 µL RNase-free water and stored until usage at −80 °C. The RNA concentration was measured using a NanoDrop 2000 spectrophotometer (Thermo Scientific, Wilmington, DE, USA). Complementary DNA (cDNA) was generated from total RNA using the High-Capacity cDNA Reverse Transcription Kit (Applied Biosystems, Foster City, CA, USA), with 2200 ng of RNA input per reaction. The resulting cDNA concentration was quantified using a NanoDrop 2000 spectrophotometer, and samples were subsequently stored at −20 °C until further use. The RT-PCR was carried out using 48-well plates and the Luminaris Color HiGreen reagents qPCR Master Mix (Thermo Scientific, Wilmington, DE, USA) 100 ng of cDNA was used for each reaction. The following genes were examined: *IL-8*, *ICAM-1*, *TIMP-2*, *MCP-1*, and *NF-kB*. The primers used for this procedure are presented in Table 2. Glyceraldehyde-3-phosphate dehydrogenase (*GAPDH*) was used as the reference housekeeping gene. The reaction conditions were set as specified by the manufacturer, and each sample was analysed in duplicate. The procedure was conducted using a StepOnePlus™ Real-Time PCR System (Thermo Scientific, Wilmington, DE, USA).

#### 4.5.2. miRNA Extraction, Reverse Transcription and Real-Time PCR

For miRNA analysis, total RNA, including small RNA fractions, was extracted using an organic phenol–chloroform method. Untreated or treated HepG2 cells were lysed in 500 µL of TRIzol reagent, then incubated for 3 h at −80 °C. Chloroform (100 μL) was added, and the samples were vigorously mixed. Phase separation was achieved by centrifugation at 12,000 rpm for 30 min at 4 °C. The aqueous phase was collected, and RNA was precipitated by adding 250 μL of isopropanol, followed by centrifugation at 12,000 rpm for 15 min. The RNA pellet was washed with 75% ethanol, air-dried, and resuspended in RNase-free water. RNA purity and concentration were assessed using a NanoDrop 2000 spectrophotometer (Thermo Fisher Scientific). Reverse transcription of mature miRNAs was performed with the TaqMan™ Advanced miRNA cDNA Synthesis Kit (Applied Biosystems). The manufacturer’s protocol included poly(A) tailing, adaptor ligation, reverse transcription, and miR-Amp pre-amplification. Specific TaqMan™ Advanced miRNA assays were used for hsa-miR-21-5p (Assay ID: 478583_mir) and the endogenous control hsa-miR-361-5p (Assay ID: 478056_mir). Quantitative real-time PCR was performed using TaqMan Fast Advanced Master Mix (Applied Biosystems) according to the recommended cycling conditions. Relative expression was quantified with the 2^−ΔΔCt^ method, using hsa-miR-361-5p as the internal reference. Untreated HepG2 cells and those treated with GO (10 μg/mL for 24 h) were analyzed to determine the effect of GO exposure on miRNA-21 expression.

#### 4.5.3. Statistical Analysis

Statistical analysis was performed using GraphPad Prism version 8. Results are presented as mean ± standard deviation (SD) from three independent experiments. Differences between groups were evaluated using one-way ANOVA, followed by Tukey’s post hoc test for multiple comparisons. Statistical significance levels are indicated as follows: * *p* < 0.05; ** *p* < 0.01; *** *p* < 0.001; **** *p* < 0.0001. *p*-value < 0.05 was considered statistically significant.

## 5. Conclusions

In this study, we successfully developed and characterized a graphene oxide-based delivery system for antisense miRNA-21 and evaluated its functional effects on HepG2 hepatocellular carcinoma cell lines. The nanosystem demonstrated good physicochemical stability, efficient loading of oligonucleotides, and pH-responsive release, which is compatible with tumor environments. GO showed good biocompatibility at working concentrations and facilitated high cellular uptake of the antisense construct, consistent with previous reports on GO’s suitability as a nanocarrier in cancer therapy [71]. However, unfunctionalized GO showed a significant impact on the expression of genes related to cellular stress. When functionalized with the antisense, miRNA-21 specifically induces a marked downregulation of key genes involved in inflammation (*IL-8, MCP-1*), cell adhesion (*ICAM-1*), fibrogenesis (*TIMP-2*), and transcription factor *NF-κB*, supporting the therapeutic relevance of miRNA-21 silencing in HCC. Notably, the observed GO-induced upregulation of miRNA-21, together with its effective suppression by the GO–antisense complex, confirms the central role of miRNA-21 modulation in driving the divergent transcriptional responses observed in this study [72]. Taken together, our results provide proof-of-concept for the application of GO-based gene delivery platforms in liver cancer therapy. We are currently conducting ongoing follow-up studies, which employing an in ovo tumor model, to bridge the gap between in vitro findings and subsequent rodent-based in vivo validation, thereby supporting a more comprehensive preclinical assessment of the proposed GO–miRNA-21 platform. This will enable a more comprehensive preclinical assessment of the GO–miRNA-21 platform, which could serve as a promising basis for future combinatorial therapies for HCC.

## Figures and Tables

**Table 1:** Kinetic parameters obtained from fitting the release profiles at pH 4.5 and pH 7.4 to the Higuchi, Korsmeyer–Peppas, and Weibull models. Reported values represent the range of fitted parameters (k_H, n, and β) and their corresponding coefficients of determination (R^2^).

Model	Parameter	pH 4.5	pH 7.4
Higuchi	k_H	0.030–0.045	0.015–0.025
	R^2^	>0.93	0.90–0.94
Korsmeyer-Peppas	n	0.32–0.41	0.28–0.36
	R^2^	0.92–0.96	0.90–0.94
Weibull	β	0.55–0.75	0.50–0.70
	R^2^	0.96–0.99	0.94–0.98

**Table 2:** Primer list of the genes considered. Abbreviations: ICAM-1: Intercellular Adhesion Molecule 1; MCP-1: Monocyte Chemoattractant Protein-1; TIMP-2: Tissue inhibitor of metalloproteinases 2; IL-8: Interleukin 8; GAPDH: Glyceraldehyde 3-phosphate dehydrogenase; NF-kB: nuclear factor kappa-light-chain-enhancer of activated B cells.

Target Gene	Forward Primer	Reverse Primer
*ICAM-1*	AGCGGCTGACGTGTGCAGTAAT	TCTGAGACCTCTGGCTTCGTCA
*MCP-1*	CCACGCAACAAATGAAGTAGCCC	CTGGAATGCTGTTCCCTTCAAG
*TIMP-2*	ACCCTCTGTGACTTCATCGTGC	GGAGATGTAGCACGGGATCATG
*IL-8*	GAGAGTGATTGAGAGTGGACCAC	CACAACCCTCTGCACCCAGTTT
*GAPDH*	TGCACCACCAACTGCTTAGC	GGCATGGACTGTGGTCATGAG
*NF-kB*	GGCAGACCAGTGTCATTGAGCA	CAGCAGAAAGCTCACCACACTC

## Data Availability

The original contributions presented in this study are included in the article. Further inquiries can be directed to the corresponding author.
